# Differentiating Enchondromas and Atypical Cartilaginous Tumors in Long Bones with Computed Tomography and Magnetic Resonance Imaging

**DOI:** 10.3390/diagnostics12092186

**Published:** 2022-09-09

**Authors:** Felix G. Gassert, Sebastian Breden, Jan Neumann, Florian T. Gassert, Christine Bollwein, Carolin Knebel, Ulrich Lenze, Rüdiger von Eisenhart-Rothe, Carolin Mogler, Marcus R. Makowski, Jan C. Peeken, Klaus Wörtler, Alexandra S. Gersing

**Affiliations:** 1 Department of Diagnostic and Interventional Radiology, School of Medicine & Klinikum rechts der Isar, Technical University of Munich, Ismaninger Strasse 22, 81675 Munich, Germany; 2 Musculoskeletal Quantitative Imaging Research Group, Department of Radiology & Biomedical Imaging, University of California San Francisco, 185 Berry St., Suite 350, San Francisco, CA 94107, USA; 3 Department of Orthopaedic Surgery, School of Medicine & Klinikum rechts der Isar, Technical University of Munich, Ismaninger Strasse 22, 81675 Munich, Germany; 4 Department of Pathology, School of Medicine & Klinikum rechts der Isar, Technical University of Munich, Ismaninger Strasse 22, 81675 Munich, Germany; 5 Department of Radiation Oncology, Klinikum rechts der Isar, Technical University of Munich, Ismaninger Strasse 22, 81675 Munich, Germany; 6 Institute of Radiation Medicine (IRM), Department of Radiation Sciences (DRS), Helmholtz Zentrum München, 85764 Munich, Germany; 7 Department of Neuroradiology, University Hospital of Munich (LMU), Marchioninistrasse 15, 81377 Munich, Germany

**Keywords:** enchondroma, atypical cartilaginous tumor, computed tomography, magnetic resonance imaging

## Abstract

The differentiation between the atypical cartilaginous tumor (ACT) and the enchondromas is crucial as ACTs require a curettage and clinical as well as imaging follow-ups, whereas in the majority of cases enchondromas require neither a treatment nor follow-ups. Differentiating enchondromas from ACTs radiologically remains challenging. Therefore, this study evaluated imaging criteria in a combination of computed tomography (CT) and magnetic resonance (MR) imaging for the differentiation between enchondromas and ACTs in long bones. A total of 82 patients who presented consecutively at our institution with either an ACT (23, age 52.7 ±18.8 years; 14 women) or an enchondroma (59, age 46.0 ± 11.1 years; 37 women) over a period of 10 years, who had undergone preoperative MR and CT imaging and subsequent biopsy or/and surgical removal, were included in this study. A histopathological diagnosis was available in all cases. Two experienced radiologists evaluated several imaging criteria on CT and MR images. Likelihood of an ACT was significantly increased if either edema within the bone (*p* = 0.049), within the adjacent soft tissue (*p* = 0.006) or continuous growth pattern (*p* = 0.077) were present or if the fat entrapment (*p* = 0.027) was absent on MR images. Analyzing imaging features on CT, the likelihood of the diagnosis of an ACT was significantly increased if endosteal scalloping >2/3 (*p* < 0.001), cortical penetration (*p* < 0.001) and expansion of bone (*p* = 0.002) were present and if matrix calcifications were observed in less than 1/3 of the tumor (*p* = 0.013). All other imaging criteria evaluated showed no significant influence on likelihood of ACT or enchondroma (*p* > 0.05). In conclusion, both CT and MR imaging show suggestive signs which can help to adequately differentiate enchondromas from ACTs in long bones and therefore can improve diagnostics and consequently patient management. Nevertheless, these features are rare and a combination of CT and MR imaging features did not improve the diagnostic performance substantially.

## 1. Introduction

Cartilaginous bone tumors are characterized by tumor cells that produce a chondroid matrix. Initially described in the late 19th century, chondrosarcomas are nowadays considered to be the most common primary malignant bone tumors and are classified into different histological grades [1,2]. Recently, chondrosarcoma grade 1 located in the appendicular skeleton was renamed into “atypical cartilaginous tumors” (ACT) according to the World Health Organization classification system [3]. ACTs permeate and entrap pre-existing trabecular bone, which is the main histopathological difference from enchondromas [3]. While former classifications only differentiated between enchondromas and central chondrosarcomas (including all grades), the current grading system considers ACTs as an intermediate lesion, due to their locally aggressive behavior [4,5]. For adequate therapeutic management, an accurate differentiation between enchondromas and ACTs in radiological imaging would be beneficial as, in the majority of the cases, enchondromas do not require any treatment, whereas ACTs require a curettage [6]. Currently, in benign cartilaginous tumors, both imaging and histopathology are helpful to exclude ACTs and increase diagnostic accuracy. Nevertheless, biopsy sample errors in heterogeneous cartilaginous tumors and histological overlap between enchondromas and ACTs lead to the major role of imaging in the diagnostic differentiation between these two subtypes of cartilaginous tumors [7]. As there is a lack of a gold standard in diagnosing cartilaginous tumors, the final diagnosis of the two entities is most commonly made taking the histopathological findings, imaging and clinical findings into consideration and is ideally performed in centers specialized in the treatment of musculoskeletal tumors [8]. Nevertheless, differentiating enchondromas from ACTs radiologically remains challenging, especially when located in the long bones [9]. Multimodal imaging including magnetic resonance (MR) imaging and conventional radiography or computed tomography (CT) is thought to be adequate for the assessment of cartilaginous tumors [10,11]. Yet, using radiographs only may be challenging for the assessment of cartilaginous tumors, since certain relevant diagnostic features may be missed, especially when differentiating between high-grade chondrosarcoma and enchondroma/ACT [11,12,13]. Additionally, some previous studies propose that nuclear medicine-based scans, e.g., scintigraphy, increase the diagnostic accuracy [14]. Yet the pathophysiological features remain similar between enchondromas and ACT and the resolution of the nuclear medicine-based scans remains insufficient for the visualization of the subtle differences between the two tumor entities [15].

The different diagnostic imaging features of ACTs, which mainly occur in the long bones more proximally (especially within the femur), and enchondromas, which most commonly appear in the proximal humerus or more distally in the long bones, have been discussed previously [9,16]. Murphey et al. evaluated differentiating criteria for enchondromas and chondrosarcomas (including former grade 1) and showed that cortical destruction, soft tissue mass, periosteal reaction and endosteal scalloping (>2/3 of cortical thickness) strongly suggested the diagnosis of chondrosarcoma. Crim et al. analyzed imaging features of cartilaginous tumors with a final histopathological diagnosis on radiographs and MR imaging and identified certain imaging features to be significant for the differentiation between enchondromas and ACTs, such as a size over 7.5 cm, presence of a soft tissue mass and cortical breakthrough [17]. A more recent study, evaluating clinical and MR imaging features for their usefulness regarding the differentiation of enchondromas and ACTs, confirmed that aside from pain, endosteal scalloping (>2/3 of cortical thickness), cortical destruction, bone expansion and presence of a soft tissue mass were useful features in order to differentiate between ACTs and enchondromas [18]. Yet, in these previous studies, there was no direct comparison between imaging features of chondroid tumors on MR and CT images. Additionally, analysis was not performed using both MR and CT imaging regarding the question of whether there is a higher accuracy if diagnostics are performed using a combination of CT and MR features. A previous study was performed in mainly appendicular bones (hand and foot), in which the analysis of MR imaging was concluded to be superior to CT regarding the differentiation between benign and malignant cartilaginous tumors [19]. Yet, this previous study only included very few patients with imaging of the long bones.

Therefore, the aim of this study was to analyze the performance and reproducibility of several features of MR and CT images for differentiating enchondromas and ACTs located in the long bones, using the final diagnosis as standard of reference.

## 2. Material and Methods

### 2.1. Patient Collective

Approval of the Institutional Review Board had been obtained prior to this study (Ethikkommitee der Technischen Universität München). Written informed consent was waived for this retrospective analysis of routinely acquired imaging and clinical data. All analyses are in line with the Declaration of Helsinki. All patients who had undergone MR imaging and CT between 2011 and 2020 and had a final diagnosis of either an enchondroma or an ACT in our interdisciplinary center for bone tumors were included in this study. For all tumors included in this analysis, histopathological diagnosis was made by one pathologist (5 years of experience) and reviewed by a further pathologist specialized in soft tissue and bone sarcomas (15 years of experience). The diagnosis was made based on the consensus of the interdisciplinary tumor board consisting of specialized pathologists, radiologists and orthopedic tumor surgeons. If patients received surgery or curettage, diagnosis was reconfirmed through postoperative histopathology of multiple-point sampling. In total, 271 patients were identified fulfilling these criteria. After evaluating imaging available in these patients, 167 patients were lost to incomplete imaging datasets. Moreover, 22 patients with enchondromas or ACTs at the hand or foot were excluded from the analyses in order to avoid a selection bias due to the different radiological and histopathologic appearance and disproportionate amount of enchondromas in these regions. Accordingly, 82 patients with the diagnosis of an enchondroma or an ACT with complete preoperative imaging datasets were included in the analysis. In none of these patients was the diagnosis changed during follow-up at our specialist center (5.8 ± 4.5 years).

### 2.2. Image Acquisition

MR and CT images which were acquired shortly before the biopsy were selected from the imaging database. MR imaging was performed on either a 1.5 or a 3 Tesla MR scanner with various protocols. All MR protocols included a T1- and a T2- weighted turbo spin echo (TSE) sequence in at least one plane, a short tau inversion recovery (STIR; either coronal or sagittal) sequence and an axial or coronal T1-weighted spin echo sequence (without and/or with fat suppression) after the administration of a contrast agent. Sequence parameters used for the acquisition are presented in the Supplementary Material.

### 2.3. Evaluation Criteria

Picture archiving and communication system (PACS) workstations were used for image analyses. Independent evaluation of images was performed by two musculoskeletal radiologists (K.W. and J.N., with 27 and 8 years of experience in musculoskeletal imaging, respectively) using a standardized scoring sheet. Both readers were blinded regarding clinical information including surgery and histopathological outcome parameters. Results of the more senior reader are given. The following features were assessed: (1) The site of the cartilaginous tumor within the skeleton (prox./dist. humerus, prox./dist. femur, others) and (2) the location within the bone (epiphysis, metaphysis, diaphysis; central or excentric) as well as (3) the maximum diameter (maximum diameter measured on MR images).

Features evaluated on MR images included (4) homogeneity of the T2 signal (homogenous or inhomogenous; where homogenous means a uniform signal of the tissue), (5) growth pattern (continuous or discontinuous; where continuous means one mass of tumor, with all tumor components connected with each other, whereas discontinuous means tumor showing two or several tumor components that are not connected with each other), (6) pattern of contrast agent uptake (none, septonodular, diffuse; as evaluated on a T1w FS sequence, where septonodular means contrast uptake in circumscribed areas with septal or nodular structure) as well as (7) presence of lobulation, (8) an extraosseous soft tissue component and edema within the (9) bone as well as the (10) surrounding soft tissue and fat entrapment as described by Vanel et al. (for each feature: yes or no) [20].

Features evaluated on CT images included (1) endosteal scalloping (none, <2/3 of cortical thickness, >2/3 of cortical thickness, cortical penetration; where scalloping means erosion of the cortical and extent refers to the relative proportion affected), (2) portion of tumor volume showing matrix calcifications (none, <1/3 of lesion volume, 1/3–2/3 of lesion volume, >2/3 of lesion volume), (3) presence of periosteal reaction and (4) expansion of bone (yes or no, for both). Additionally, image quality was assessed for both CT and MR images using a four-point scale (1 = poor, 2 = moderate, 3 = good, 4 = excellent) based on the reader’s experience. For evaluation of the intrareader agreement, one radiologist (J.N.) performed a second reading of all patients once again after four weeks, blinded to the previous results. Besides individual features, combinations of all MR and CT features as well as combinations of features with a sensitivity >0.85 and criteria with a specificity >0.85 were evaluated.

### 2.4. Statistical Analysis

All statistical analyses were performed by a resident supported by a senior biomedical statistician using the statistical package R version 3.2.4 (R Foundation for Statistical Computing, Vienna, Austria). All statistical tests were performed two-sided with a level of significance (α) of *p* < 0.05. The frequencies of MR imaging findings and demographic parameters were compared between groups with crosstabs and Pearson’s chi-squared test and Fisher’s exact test, respectively, for binary parameters. Logistic regression models were used to estimate the likelihood of the presence of certain morphological features for the diagnosis of an ACT. Additionally, an analysis of combinations of all features showing a sensitivity higher than 0.85 with features showing a specificity higher than 0.85 was performed. A receiver operating characteristic (ROC) analysis was used to assess the performance of the parameter “maximum diameter” for the differentiation between ACTs and enchondromas. Youden’s J statistic was used to identify the optimal cut-off value. Fleiss’ κ was used for evaluation of the intra- and inter-reader agreement of CT and MR imaging findings. If differing, values are given for the more senior reader.

## 3. Results

### 3.1. Patient Characteristics

Of 82 cartilaginous tumors analyzed, 59 (72%) were diagnosed as enchondroma and 23 (28%) as ACT using the interdisciplinary diagnosis as standard of reference. Mean age of all study patients was 47.88 ± 13.89 (48 median; min/max 16–76) years and there was no significant difference in age between patients with ACTs and those with enchondromas (*p* = 0.12). Moreover, there was no significant difference regarding the sex distribution between the patient group with ACTs and the group with enchondromas (ACT, 60.9% women; enchondroma 62.5% women; *p* = 0.63).

### 3.2. CT and MR Image Quality

Image quality was rated as excellent or good in 76.8% of the MR images and 91.5% of the CT images. In 23.2% of the MR images and 8.5% of the CT images the image quality was rated as moderate.

### 3.3. Tumor Localization and Size

The majority of tumors was located at either the proximal humerus (34, 41.5%) or the distal femur (24, 29.3%), whereas fewer tumors were located in other regions (24, 29.3%). Furthermore, localization of the tumor within the bone was analyzed and showed no significant influence on probability of the final diagnosis of an ACT (Supplementary Material). Moreover, there was no significant difference seen regarding the side of occurrence of the tumor (ACT, 43.5% right side; enchondroma, 49.2% right side; *p* = 0.82).

Additionally, the difference between centrally located and eccentrically located tumors within the bone was assessed: 28.6% of the ACTs and 17.2% of the enchondromas were located eccentrically within the bone, resulting in an increase of the likelihood of the diagnosis of an ACT by the factor of 1.91 if located eccentrically within the bone (*p* = 0.34) (Table 1).

**Figure 1:**
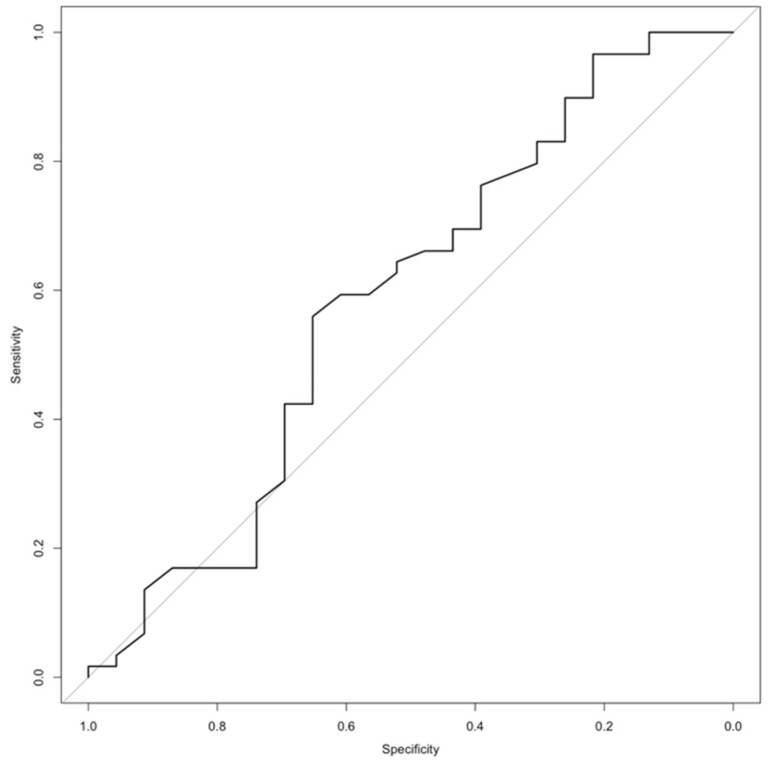
Receiver-operating-characteristic (ROC) curve demonstrating the association between maximum tumor diameter and entity (AUC 0.585, 95%-confidence interval 0.434–0.735).

For the evaluation of the tumor diameter and the tumor subtype, ROC analysis was performed, resulting in an AUC of 0.59 for differentiating between ACTs and enchondromas using an optimal cut-off value of 4.7 cm (95% CI 0.43–0.74 cm) as shown in Figure 1 (sensitivity 0.65, specificity 0.56). Applying this cut-off value, there was no significant influence of the tumor size on the likelihood of a tumor being an ACT (OR: 2.34, 95% CI 0.78–6.71, *p* = 0.14).

### 3.4. CT and MR Imaging Criteria

**Figure 2:**
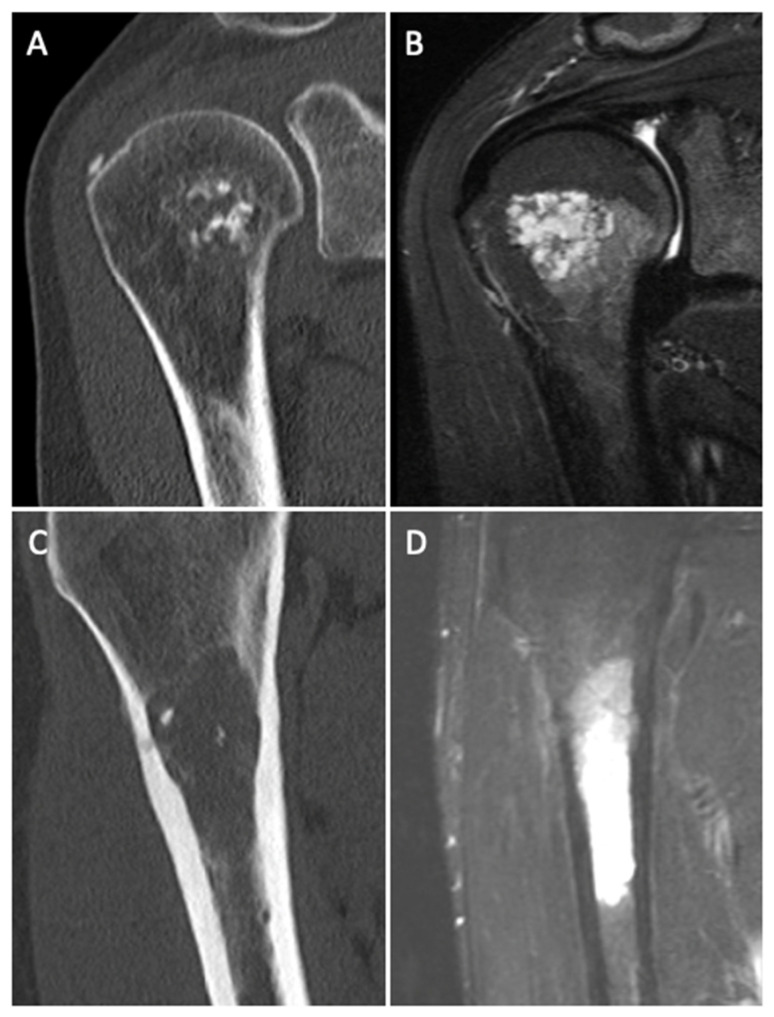
Exemplary computed tomography (**A**,**C**) and magnetic resonance images (short-tau inversion recovery (STIR) image) (**B**,**D**) of an enchondroma in the coronal plane (**A**,**B**) showing a lobulated tumor architecture without endosteal scalloping and an ACT in the sagittal plane showing endosteal scalloping and edema within the bone (**C**,**D**).

Besides tumor localization and diameter, several CT and MR imaging criteria and their value for the differentiation between ACTs and enchondromas were analyzed. Exemplary CT and MR images for an enchondroma and an ACT are shown in Figure 2.

ACTs showed a higher rate of tumors with a continuous growth pattern than enchondromas (68.2% vs. 25.4%) with an increase in the likelihood of the diagnosis of an ACT by a factor of 6.06 (95% CI 2.12–18.96, *p* < 0.001) when the growth pattern of the tumor was continuous. Presence of matrix calcifications in general did not show any significant change in odds ratio between the two groups (*p* = 1). Nevertheless, if matrix calcifications were present in less than 1/3 of the tumor, the likelihood of the diagnosis of an ACT was increased by a factor of 3.75 (95% CI 1.38–10.77, *p* = 0.011) with 66.7% of ACTs and 28.8% of enchondromas showing matrix calcifications in less than 1/3 of the tumor. Choosing a cutoff at 2/3 of tumor calcified, there was no significant difference in odds ratio observed (*p* = 0.68). Moreover, periosteal reaction was seen in 34.8% of ACTs and 3.3% of enchondromas, increasing the likelihood of the diagnosis of an ACT by a factor of 13.87 (95% CI 3.01–109.46, *p* < 0.001) if a periosteal reaction was present. Furthermore, 95.7% of the ACTs and 74.6% of the enchondromas caused endosteal scalloping, increasing the likelihood of the diagnosis of an ACT by the factor of 6.25 (95% CI 1.19–166.18, *p* = 0.0327) if endosteal scalloping was seen. Presence of endosteal scalloping of more than 2/3 of cortical thickness was observed in 65.2% of ACTs and 10.2% of enchondromas and therefore increased the likelihood of an ACT by a factor of 15.48 (95% CI 4.86–56.7, *p* < 0.001). Cortical penetration was present in 26.1% of the ACTs and 1.7% of the enchondromas, resulting in an odds ratio of 17.68 (95% CI 2.66–480.45, *p* = 0.002) or presence of cortical penetration. Of the ACTs, 43.5% showed expansion whereas 18.6% of the enchondromas were associated with expansion of bone resulting in an increase of the likelihood of the diagnosis of an ACT by a factor of 3.29 (95% CI 1.13–9.73, *p* = 0.027) if the tumor caused bone expansion.

The tumor tissue showed a homogenous T2 signal on MR imaging in 31.6% of the ACTs and 28.3% of the enchondromas with no significant influence on the likelihood of an ACT or enchondroma (OR 1.18; 95% CI 0.34–3.76, *p* = 0.77). Moreover, lobulation of the tumor had no significant influence on the likelihood of being an ACT or enchondroma with all ACTs showing a lobulated architecture and only one enchondroma without lobulation (OR 1.21, 95% CI 0.05–30.66, *p* = 0.91). All lesions analyzed showed a septonodular contrast agent uptake (*p* = 1.00).

**Figure 3:**
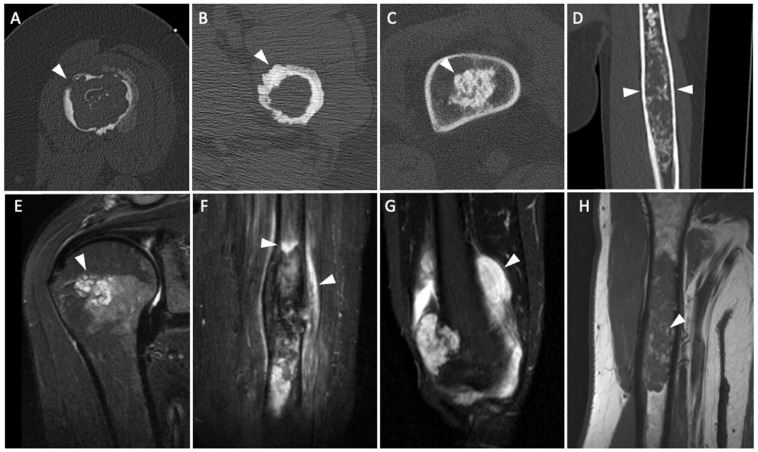
Exemplary computed tomography images demonstrating the features endosteal scalloping, cortical penetration (**A**)**,** periosteal reaction (**B**), Calcifications >2/3 (**C**) and Expansion of the bones (**D**) as well as magnetic resonance (MR) images for discontinuous growth pattern (short-tau inversion recovery (STIR) image) (**E**), edema within the bone and adjacent soft tissue (short-tau inversion recovery (STIR) image) (**F**), extraosseous soft tissue component (short-tau inversion recovery (STIR) image) (**G**) and fat entrapment (T1 weighted image) (**H**). Features are highlighted by white arrow heads.

Presence of an extraosseous soft tissue component increased the likelihood of an ACT by a factor of 20.32 (95% CI 1.01–410.31, *p* = 0.0495) as it was observed in 13.0% of the ACTs and in none of the enchondromas. Edema within both the bone and the adjacent soft tissue was more common in ACTs than in enchondromas with 17.4% (bone) and 26.1% (soft tissue) of the ACTs and 0.0% (bone) and 1.7% (soft tissue) of the enchondromas showing edema. Therefore, the likelihood of the diagnosis of an ACT was increased by a factor of 27.46 (95% CI 1.41–533.26, *p* = 0.029) if edema was detected within the adjacent bone and by a factor of 17.68 (95% CI 2.66–480.45, *p* = 0.002) if there was edema within the adjacent soft tissue. The presence of fat entrapment significantly decreased the likelihood of an ACT by a factor of 0.29 (95% CI 0.10–0.83, *p* = 0.027). Exemplary images for significant features are shown in Figure 3. The inter-observer reliability for the radiologists was substantial to almost perfect for all individual criteria evaluated on both CT and MR images (κ = 0.75–1.00) and the intra-observer reliability was excellent (κ = 0.85–0.96), respectively. The inter-observer reliability of the pathologists was excellent for the final diagnosis (κ = 0.97).

Results of the analysis of combined MR and CT features are displayed in the Supplementary Material. No substantial increase in Odds ratio was observed for combinations of features as compared to individual criteria. In an additional analysis of combinations of all features showing high sensitivity with features showing high specificity no combination showing both high sensitivity and high specificity was observed. Results are displayed in the Supplementary Material.

## 4. Discussion

The differentiation between ACTs and enchondromas is crucial as ACTs require a curettage and clinical as well as imaging follow-ups, whereas in the majority of cases enchondromas neither require a treatment, unless they are symptomatic, nor require clinical and imaging follow-ups. Therefore, in this study, we evaluated the diagnostic value of several criteria for differentiation between enchondromas and ACTs in long bones, on both MR and CT images individually as well as the combination of different features of these techniques.

Although previous studies state, that chondrosarcomas (including former grade 1) occur more often in older patients compared to enchondromas, and we observed a similar trend comparing ACTs and enchondromas, in this study as well as in previously published studies, no significant difference in age between patients with ACTs and enchondromas could be observed [21,22].

As known from previous studies, the majority of tumors is located in the proximal humerus or the distal femur [9,11,23]. Although previous studies showed that enchondromas are more frequently found in the diaphysis of long tubular bones and that the metaphysis is more commonly involved in ACTs (including former grade 1), no significant difference between the distribution of entities within dia-, meta- or epiphysis was found in this study including ACTs and enchondromas only [11,21]. Although there was no significant difference found, the average diameter of ACTs was larger than that of enchondromas, emphasizing the findings of previous studies, in which ACTs showed larger tumor diameters compared to enchondromas [24,25].

Besides rather obvious features such as the localization and tumor size, several imaging parameters have previously shown to enable the differentiation between ACTs and enchondromas. In histopathology, ACTs are likely to show a more aggressive growth pattern, which can cause typical cortical thinning on radiographs and on CT, as well as indirect signs of rapid and aggressive growth, such as destruction, permeation and entrapment of pre-existing lamellar bone trabeculae [3]. These changes in the more aggressive ACT can be visualized in MR and CT imaging. Murphey et al. tried to define MR imaging criteria for differentiation between enchondromas and ACTs based on these pathological changes, including endosteal scalloping, interrupted cortices, periosteal reaction or presence of a soft tissue mass [21]. Douis et al. were the last to analyze differences of ACTs and enchondromas and showed that the presence of edema within both the bone and the adjacent soft tissue as well as an extraosseous soft tissue component were significantly more likely to occur in ACTs [18]. Although these criteria were rarely observed in our series, they significantly increased the likelihood of the diagnosis of an ACT, showing the biggest increase in likelihood of an ACT among all analyzed imaging criteria. Fat entrapment—another parameter identified on MR-images—was initially described by Vanel et al. and, similar to previous studies, we also observed that presence of fat entrapment was higher in enchondromas as compared to ACTs and significantly decreased the likelihood of ACTs [4,18].

CT is known to even better evaluate some bony changes, such as endosteal scalloping, periosteal reaction and matrix calcifications [26].

Endosteal scalloping, which refers to the focal resorption of the inner layer of the cortex of the involved bone can occur in both enchondromas and ACTs. Nevertheless, according to Douis et al., endosteal scalloping of more than 2/3 of cortical thickness is the most sensitive imaging parameter for the diagnosis of an ACT as it is likely to be caused by the lobular growth pattern of ACT as seen in histopathology [3,18].

Although the presence of endosteal scalloping has significantly increased the likelihood of an ACT in our study, especially if the tumor showed full cortical penetration, there was also one case of cortical penetration observed in an enchondroma. The degree of endosteal scalloping is a strong indicator of whether an ACT is present as most cases of ACTs showed this feature. Nevertheless, there was one case of an ACT without presence of endosteal scalloping.

Although only approximately one third of the ACTs showed periosteal reactions, this finding is a strong indicator for the diagnosis of an ACT as a sign of its aggressive growth. These results are in line with a study by Douis et al. who also showed a higher proportion of periosteal reaction in ACTs compared to enchondromas using MR images for the evaluation of a periosteal reaction [18].

Interestingly, almost all tumors of both entities showed at least some matrix calcifications. Although a study by Errani et al. demonstrated a strong tendency towards a higher rate of calcifications in enchondromas as compared to ACTs, there was no study so far that showed significant differences in matrix calcifications between ACTs and enchondromas [23].

In our study on the other hand, possibly due to a relatively high number of patients and the use of CT in all cases, a significant increase of likelihood of the diagnosis of an ACT was observed if matrix calcifications were present in less than 1/3 of the tumor tissue, which on a histopathological level can be explained through less aggressive growth of enchondroma with rather degenerative features such as necrosis and calcifications [3].

Of the criteria examined on MR images, especially edema within the bone and adjacent soft tissue, the growth pattern and the extraosseous soft tissue component are most relevant based on the results of this study. Of the criteria evaluated on CT images, extent of calcifications and endosteal scalloping as well as presence of bone expansion and a periosteal reaction showed a significant increase in likelihood for the diagnosis of an ACT and therefore could be helpful for the differentiation between ACTs and enchondromas.

As none of the individual features showed both high sensitivity and high specificity, additional analysis of a combination of all CT-features with all MR-features and all features showing a high sensitivity with all features showing high specificity was performed. A previous study in mainly appendicular bones (hand and foot) concluded MR imaging to be superior to CT regarding the differentiation between benign and malignant cartilaginous tumors [19]. In this analysis, including enchondromas and ACTs located in long bones, no increase in odds ratio could be observed for combination of CT and MR images. Additionally, none of the combinations of criteria showed both a high sensitivity and a high specificity for the same analysis.

**Figure 4:**
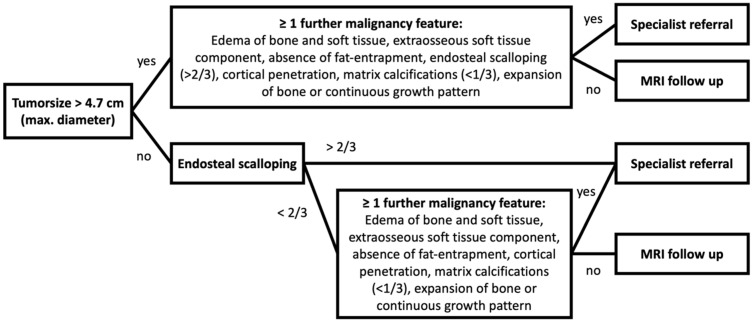
Flow chart for diagnostic procedure based on imaging findings in MRI and CT.

We showed that the presence of edema within the bone and adjacent soft tissue, an extraosseous soft tissue component, absence of fat entrapment, endosteal scalloping of more than 2/3 of original cortical thickness and cortical penetration, matrix calcifications in less than 1/3 of the tumor volume, expansion of bone and a continuous growth pattern significantly increased the likelihood of an ACT as compared to an enchondroma significantly. Consequently, especially in larger tumors with additionally at least one of these mentioned features the patient should be referred to a specialist for a potential biopsy and/or surgical removal. If the tumor is smaller in size and none of these features are present, watch and wait can be considered as an option—yet, if a change in morphology or size of the tumor is detected the patient should be referred to a specialist for further diagnostics. Based on those findings we implemented a flow chart for possible diagnostic procedure (Figure 4). Biopsy in general should be taken from the region with the most malignant impression based on imaging features and if an extraosseous soft tissue component is present one should obtain a sample from this component if anatomically feasible.

This study has limitations. Some of the features increasing the likelihood of an ACT only occur rarely. Although the increase of likelihood was significant, if an extraosseous soft tissue component or edema within the bone or adjacent soft tissue was present, results are therefore based on small numbers of cases presenting these features. A combination of histopathological analysis, clinical findings and radiologic image interpretation was used as standard of reference for this study. Therefore, the standard of reference might be biased due to inclusion of imaging criteria. Additionally, in patients diagnosed with biopsy, sampling errors may occur, although biopsies were performed from the regions appearing most malignant in imaging in order to avoid these sampling errors. Overall, this is a common approach in comparable studies as there is no specific immunohistochemical analysis available for the differentiation between ACTs and enchondromas [11,18,21]. Furthermore, novel CT and MR imaging analysis techniques such as texture analysis, which have been shown to support the differentiation between several types of malignancies, were not applied in this study [27]. This method has been shown to be able to discriminate between ACTs and enchondromas by a variety of texture parameters [28,29], but may not yet be available to all radiologists and physicians. Additionally, clinical parameters such as pain and nuclear medicine techniques such as scintigraphy have been shown to be useful for differentiation between high grade chondrosarcomas and enchondromas/ACTs. Nevertheless, these techniques did not seem to be beneficial regarding the differentiation of ACTs and enchondromas [14,15]. Further studies in larger cohorts using these new techniques in order to differentiate ACTs and enchondromas will be needed in the future. Nevertheless, the results of this study are intended to support the radiologists in daily routine in diagnosing ACTs and enchondromas and are therefore based on common clinical imaging techniques. Based on the fact that a combination of imaging parameters did not substantially improve the diagnostic performance, proposition of a prediction model based upon imaging finding does not seem feasible.

In conclusion, this study shows that both CT and MR imaging features, such as the presence of edema within the bone and adjacent soft tissue, extraosseous soft tissue components, endosteal scalloping, matrix calcifications in less than 1/3 of tumor tissue, expansion of bone and a continuous growth pattern, are able to significantly help for the differentiation between ACTs and enchondromas located in long bones. A combination of these features did not improve the diagnostic performance substantially. Therefore, from a clinical point of view both of these techniques can be useful in the initial diagnosis of cartilaginous tumors.

## Figures and Tables

**Table 1:** Analysis of features assessed on MR and CT images.

Variable		ACT	Ench.	Odds Ratio (95% CI)	*p*-Value	Sensitivity	Specificity	PPV	NPV
Location in bone	excentric	6	10	1.91 (0.56–6.19)	0.343	0.286	0.828	0.375	0.762
	central	15	48						
Diameter	>Average	9	17	1.58 (0.56–4.38)	0.432	0.391	0.712	0.346	0.75
	<Average	14	42						
Diameter (optim)	>4.7 cm	15	26	2.34 (0.87–6.71)	0.139	0.652	0.559	0.366	0.805
	<4.7 cm	8	33						
Growth pattern	Continuous	15	15	6.06	<0.001	0.682	0.746	0.5	0.863
	Discontinuous	7	44						
T2 Signal	Homogenous	6	13	1.18 (0.34–3.76)	0.774	0.316	0.717	0.316	0.717
	Inhomogenous	13	33						
Lobulation	Yes	23	58	1.21 (0.05–30.66)	0.91	1	0.017	0.284	1
	No	0	1						
Fat-entrapment	Yes	12	47	0.29 (0.10–0.83)	0.027	0.522	0.203	0.203	0.522
	No	11	12						
Matrix calcifications	No	1	2	1.37 (0.04–17.71)	1	0.044	0.966	0.333	0.722
	Yes (any)	22	57						
Matrix calcifications	<1/3	14	17	3.75 (1.38–10.77)	0.011	0.609	0.712	0.452	0.824
	>1/3	9	42						
Matrix calcifications	<2/3	20	54	0.61 (0.13–3.41)	0.68	0.870	0.085	0.27	0.625
	>2/3	3	5						
Periosteal reaction	Yes	8	2	13.87 (3.01–109.46)	<0.001	0.348	0.966	0.8	0.792
	No	15	57						
Expansion	Yes	10	11	3.29 (1.13–9.73)	0.027	0.435	0.814	0.476	0.787
	No	13	48						
Endosteal scalloping	Yes (any)	22	44	6.25 (1.19–166.18)	0.0327	0.957	0.254	0.333	0.938
	No	1	15						
Endosteal scalloping	>2/3	15	6	15.48 (4.86–56.7)	<0.001	0.652	0.898	0.714	0.869
	<2/3	8	53						
Endosteal scalloping	Penetration	6	1	17.68 (2.66–480.45)	0.002	0.261	0.983	0.857	0.773
	No Penetration	17	58						
Extraosseous soft tissue component	Yes	3	0	20.32 (1.01–410.31)	0.0495	0.13	1	1	0.747
	No	20	59						
Edema bone	Yes	4	0	27.46 (1.41–533.26)	0.029	0.174	1	1	0.756
	No	19	59						
Edema soft tissue	Yes	6	1	17.68 (2.66–480.45)	0.002	0.261	0.983	0.857	0.773
	No	17	58						

Odds ratio, Sensitivity, Specificity, PPV and NPV given for the identification of ACT versus enchondroma, respectively. PPV = positive predictive value; NPV = negative predictive value; CI = confidence interval. Values are indicated for the more senior reader.

## Data Availability

The datasets used and/or analyzed during the current study are available from the corresponding author on reasonable request.

## Supplementary Materials

The following supporting information can be downloaded at: https://www.mdpi.com/article/10.3390/diagnostics12092186/s1, Table S1: Magnetic resonance imaging parameters; Table S2 Region of tumor localization; Table S3 Tumor localization within the bone; Table S4 Analysis of combined MR and CT features; Table S5 Analysis of combinations of features with high sensitivity and features with high specificity.

## Abbreviations

ACT	Atypical cartilaginous tumor
AUC	Area under the curve
CI	Confidence interval
CT	Computed tomography
FS	Fat saturation
GD	Gadolinium
IM	Intermediate-weighted
MR	Magnetic resonance
NPV	Negative predictive value
OR	Odds ratio
PACS	Picture archiving and communication system
PPV	Positive predictive value
ROC	Receiver operating characteristic
STIR	Short tau inversion recovery
TSE	Turbo spin echo

## References

[1] Thorkildsen J., Taksdal I., Bjerkehagen B., Haugland H.K., Børge Johannesen T., Viset T., Norum O.J., Bruland Ø., Zaikova O. (2019). Chondrosarcoma in Norway 1990–2013; an epidemiological and prognostic observational study of a complete national cohort. Acta Oncol..

[2] Van Praag Veroniek V.M., Rueten-Budde A.J., Ho V., Dijkstra P.D.S., Fiocco M., van de Sande M.A.J. (2018). Incidence, outcomes and prognostic factors during 25 years of treatment of chondrosarcomas. Surg. Oncol..

[3] WHO Classification of Tumours Editorial Board (2020). World Health Organization Classification of Soft Tissue and Bone Tumours.

[4] Deckers C., Steyvers M.J., Hannink G., Schreuder H.W.B., de Rooy J.W.J., Van Der Geest I.C.M. (2020). Can MRI differentiate between atypical cartilaginous tumors and high-grade chondrosarcoma? A systematic review. Acta Orthop..

[5] Jo V.Y., Fletcher C.D. (2014). WHO classification of soft tissue tumours: An update based on the 2013 (4th) edition. Pathology.

[6] Dierselhuis E.F., Gerbers J.G., Ploegmakers J.J., Stevens M., Suurmeijer A.J., Jutte P.C. (2016). Local Treatment with Adjuvant Therapy for Central Atypical Cartilaginous Tumors in the Long Bones: Analysis of Outcome and Complications in One Hundred and Eight Patients with a Minimum Follow-up of Two Years. J. Bone Jt. Surg. Am. Vol..

[7] Laitinen M.K., Stevenson J.D., Parry M.C., Sumathi V., Grimer R.J., Jeys L.M. (2018). The role of grade in local recurrence and the disease-specific survival in chondrosarcomas. Bone Jt. J..

[8] Lalam R., Bloem J.L., Noebauer-Huhmann I.M., Wörtler K., Tagliafico A., Vanhoenacker F., Nikodinovska V.V., Sanal H.T., Woude H.V., Papakonstantinou O. (2017). ESSR Consensus Document for Detection, Characterization, and Referral Pathway for Tumors and Tumorlike Lesions of Bone. Semin. Musculoskelet. Radiol..

[9] Deckers C., Schreuder B.H., Hannink G., de Rooy J.W., van der Geest I.C. (2016). Radiologic follow-up of untreated enchondroma and atypical cartilaginous tumors in the long bones. J. Surg. Oncol..

[10] Geirnaerdt M.J., Hermans J., Bloem J.L., Kroon H.M., Pope T.L., Taminiau A.H., Hogendoorn P.C. (1997). Usefulness of radiography in differentiating enchondroma from central grade 1 chondrosarcoma. AJR Am. J. Roentgenol..

[11] Choi B.B., Jee W.H., Sunwoo H.J., Cho J.H., Kim J.Y., Chun K.A., Hong S.J., Chung H.W., Sung M.S., Lee Y.S. (2013). MR differentiation of low-grade chondrosarcoma from enchondroma. Clin. Imaging.

[12] Kendell S.D., Collins M.S., Adkins M.C., Sundaram M., Unni K.K. (2004). Radiographic differentiation of enchondroma from low-grade chondrosarcoma in the fibula. Skelet. Radiol..

[13] Nascimento D., Suchard G., Hatem M., de Abreu A. (2014). The role of magnetic resonance imaging in the evaluation of bone tumours and tumour-like lesions. Insights Imaging.

[14] Higuchi T., Taki J., Sumiya H., Kinuya S., Nakajima K., Namura M., Tonami N. (2005). Characterization of cartilaginous tumors with 201Tl scintigraphy. Ann. Nucl. Med..

[15] Kaya G.C., Demir Y., Ozkal S., Sengoz T., Manisali M., Baran O., Koc M., Tuna B., Ozaksoy D., Havitcioglu H. (2010). Tumor grade-related thallium-201 uptake in chondrosarcomas. Ann. Nucl. Med..

[16] Deckers C., de Leijer E.M., Flucke U., de Rooy J.W.J., Schreuder H.W.B., Dierselhuis E.F., van der Geest I.C.M. (2021). Curettage and cryosurgery for enchondroma and atypical cartilaginous tumors of the long bones: Oncological results of a large series. J. Surg. Oncol..

[17] Crim J., Schmidt R., Layfield L., Hanrahan C., Manaster B.J. (2015). Can imaging criteria distinguish enchondroma from grade 1 chondrosarcoma?. Eur. J. Radiol..

[18] Douis H., Parry M., Vaiyapuri S., Davies A.M. (2018). What are the differentiating clinical and MRI-features of enchondromas from low-grade chondrosarcomas?. Eur. Radiol..

[19] Lee S., Yoon M.A. (2022). Assessment of central cartilaginous tumor of the appendicular bone: Inter-observer and intermodality agreement and comparison of diagnostic performance of CT and MRI. Acta Radiol..

[20] Vanel D., Kreshak J., Larousserie F., Alberghini M., Mirra J., De Paolis M., Picci P. (2013). Enchondroma vs. chondrosarcoma: A simple, easy-to-use, new magnetic resonance sign. Eur. J. Radiol..

[21] Murphey M.D., Flemming D.J., Boyea S.R., Bojescul J.A., Sweet D.E., Temple H.T. (1998). Enchondroma versus chondrosarcoma in the appendicular skeleton: Differentiating features. Radiographics.

[22] Flemming D.J., Murphey M.D. (2000). Enchondroma and chondrosarcoma. Semin. Musculoskelet. Radiol..

[23] Errani C., Tsukamoto S., Ciani G., Akahane M., Cevolani L., Tanzi P., Kido A., Honoki K., Tanaka Y., Donati D.M. (2017). Risk factors for local recurrence from atypical cartilaginous tumour and enchondroma of the long bones. Eur. J. Orthop. Surg. Traumatol..

[24] Delling G., Jobke B., Burisch S., Werner M. (2005). Cartilage tumors. Classification, conditions for biopsy and histologic characteristics. Orthopade.

[25] Engel H., Herget G.W., Füllgraf H., Sutter R., Benndorf M., Bamberg F., Jungmann P.M. (2020). Chondrogenic Bone Tumors: The Importance of Imaging Characteristics. RöFo-Fortschritte auf dem Gebiet der Röntgenstrahlen und der Bildgebenden Verfahren.

[26] Manaster B.J., Dalinka M.K., Alazraki N., Berquist T.H., Daffner R.H., DeSmet A.A., el-Khoury G.Y., Goergen T.G., Keats T.E., Newberg A. (2000). Follow-up examinations for bone tumors, soft tissue tumors, and suspected metastasis post therapy. American College of Radiology. ACR Appropriateness Criteria. Radiology.

[27] Csutak C., Ștefan P.A., Lenghel L.M., Moroșanu C.O., Lupean R.A., Șimonca L., Mihu C.M., Lebovici A. (2020). Differentiating High-Grade Gliomas from Brain Metastases at Magnetic Resonance: The Role of Texture Analysis of the Peritumoral Zone. Brain Sci..

[28] Lisson C.S., Lisson C.G., Flosdorf K., Mayer-Steinacker R., Schultheiss M., von Baer A., Barth T.F.E., Beer A.J., Baumhauer M., Meier R. (2018). Diagnostic value of MRI-based 3D texture analysis for tissue characterisation and discrimination of low-grade chondrosarcoma from enchondroma: A pilot study. Eur. Radiol..

[29] Fritz B., Müller D.A., Sutter R., Wurnig M.C., Wagner M.W., Pfirrmann C.W.A., Fischer M.A. (2018). Magnetic Resonance Imaging-Based Grading of Cartilaginous Bone Tumors: Added Value of Quantitative Texture Analysis. Investig. Radiol..

