# Supplementary material for: Differentiating Enchondromas and Atypical Cartilaginous Tumors in Long Bones with Computed Tomography and Magnetic Resonance Imaging
Source: Diagnostics (Basel). 2022 Sep 9;12(9):2186. doi: 10.3390/diagnostics12092186 (PMC9497620; doi:10.3390/diagnostics12092186)
Supplement: Supplementary file 1 [file diagnostics-12-02186-s001.zip › diagnostics-1852608-supplementary.pdf]

Supplementals

Table S1. Magnetic resonance imaging parameters.

Supplementary Table: Magnetic Resonance imaging sequence parameters

	Knee							
Sequence	STIR		T_1 -/+ GD		T_2		T_1-FS-GD	
Field strength (T)	1.5	3.0	1.5	3.0	1.5	3.0	1.5	3.0
Repetition time (ms)	4670	2850	557	1100	4020	4260	595	1050
Echo time (ms)	69	47	15	12	104	94	14	13
Flip angle (°)	180	160	90	180	180	180	90	180
Field of view (mm)	220	180	220	200	160	160	160	160
In-plane resolution (mm)	0.7x 0.7	0.6x 0.6	0.5x 0.5	0.5x 0.5	0.5x 0.5	0.5x 0.5	0.6x 0.6	0.6x 0.6
Slice thickness (mm)	4	4	4	3	4	4	4	4
Gap (%)	50	25	25	20	25	10	25	10
Bandwidth (Hz/pixel)	130	252	90	172	111	200	94	150
Echo train length (n)	24	20		183	14	20		288

	Shoulder							
Sequence	IM-FS	STIR	T_1 -/+ GD		T_2		T_1-FS-GD	
Field strength (T)	1.5	3.0	1.5	3.0	1.5	3.0	1.5	3.0
Repetition time (ms)	3950	4500	537	1040	4300	4990	500	722
Echo time (ms)	45	47	16	12	89	78	16	12
Flip angle (°)	180	156	90	175	180	180	90	180
Field of view (mm)	160	180	160	160	180	180	160	180
In-plane resolution (mm)	0.2x 0.2	0.6x 0.6	0.2x 0.2	0.5x 0.4	0.4x 0.4	0.6x 0.6	0.3x 0.3	0.6x 0.6
Slice thickness (mm)	3	4	3	3	3	4	3	4
Gap (%)	10	25	10	10	20	20	20	20
Bandwidth (Hz/pixel)	109	252	85	161	109	200	85	150
Echo train length (n)	33	17		262	58	15		203

	Hip/Pelvis							
Sequence	STIR		T_1 -/+ GD		T_2		T_1-FS-GD	
Field strength (T)	1.5	3.0	1.5	3.0	1.5	3.0	1.5	3.0
Repetition time (ms)	3470	9520	542	855	6110	5600	522	775
Echo time (ms)	31	44	15	12	85	87	13	12
Flip angle (°)	150	144	172	180	180	180	180	136
Field of view (mm)	260	350	260	350	180	350	180	350
In-plane resolution (mm)	0.8x 0.8	0.9x 0.9	0.4x 0.4	0.8x 0.7	0.6x 0.6	0.7x 0.7	0.6x 0.6	0.7x 0.7
Slice thickness (mm)	4	5	4	5	5	5	5	5
Gap (%)	60	40	20	50	60	40	60	40
Bandwidth (Hz/pixel)	161	246	128	160	119	203	130	160
Echo train length (n)	19	24	118	162	19	37	118	116

Inversion time (ms)	155	210	210	150	210

STIR: short tau inversion recovery; GD: gadolinium; FS: fat saturation; IM: intermediate-weighted

Table S2. Region of tumor localization.

	Prox. Humerus	Prox. Femur	Dist. Femur	Prox. + dist. Humerus	Others
ACT	3	6	10	0	4
Enchondroma	31	2	14	2	10

Table S3. Tumor localization within the bone.

	Epiphysis	Epi-Metaphysis	Metaphysis	Meta-Diaphysis	Diaphysis	All three
ACT	1	3	5	5	4	2
Enchondrom	1	11	16	13	14	2

Table S4. Analysis of combined MR and CT features.

Combined features CT + MR		ACT	Ench.	Odds ratio (95% CI)	P-value	Sensitivity	Specificity	PPV	NPV
Cont. growth pattern	Yes	7	3	7.71 (1.86–41.42)	0.004	0.304	0.949	0.7	0.778
+ Expansion	No	16	56						
Cont. growth pattern	Yes	12	8	6.69 (2.24–21.47)	0.001	0.522	0.864	0.6	0.823
+ Matr. Calcifications <1/3	No	11	51						
Cont. growth pattern	Yes	15	14	6.62 (2.3–20.89)	<0.001	0.682	0.763	0.517	0.865
+ Matr. Calcifications <2/3	No	7	45						
Cont. growth pattern	Yes	15	12	8.03 (2.75–25.9)	<0.001	0.682	0.797	0.556	0.87
+ Endosteal scalloping (any)	No	7	47						
Cont. growth pattern	Yes	12	4	14.0 (4.02–60.01)	<0.001	0.522	0.932	0.75	0.833
+ Endosteal scalloping > 2/3	No	11	55						
Cont. growth pattern	Yes	6	0	44.2 (2.37–824.01)	0.011	0.261	1	1	0.776
+ Penetration	No	17	59						
Cont. growth pattern	Yes	7	0	57.58 (3.12–1064.23)	0.007	0.318	1	1	0.797
+ Periosteal reaction	No	15	59						
Extraoss. soft tissue comp.	Yes	2	0	13.84 (0.64–299.94)	0.094	0.087	1	1	0.737
+ Expansion	No	21	59						
Extraoss. soft tissue comp.	Yes	3	0	20.32 (1.01–410.31)	0.05	0.13	1	1	0.747
+ Matr. Calcifications <1/3	No	20	59						
Extraoss. soft tissue comp.	Yes	3	0	20.32 (1.01–410.31)	0.05	0.13	1	1	0.747
+ Matr. Calcifications <2/3	No	20	59						
Extraoss. soft tissue comp.	Yes	3	0	20.32 (1.01–410.31)	0.05	0.13	1	1	0.747

+ Endosteal scalloping (any)	No	20	59						
Extraoss. soft tissue comp.<br>+ Endosteal scalloping > 2/3	Yes<br>No	3<br>20	0<br>59	20.32 (1.01–410.31)	0.05	0.13	1	1	0.747
Extraoss. soft tissue comp.<br>+ Penetration	Yes<br>No	3<br>20	0<br>59	20.32 (1.01–410.31)	0.05	0.13	1	1	0.747
Extraoss. soft tissue comp.<br>+ Periosteal reaction	Yes<br>No	3<br>20	0<br>59	20.32 (1.01–410.31)	0.05	0.13	1	1	0.747
Edema bone<br>+ Expansion	Yes<br>No	3<br>20	0<br>59	20.32 (1.01–410.31)	0.05	0.13	1	1	0.747
Edema bone<br>+ Matr. Calcifications <1/3	Yes<br>No	3<br>20	0<br>59	20.32 (1.01–410.31)	0.05	0.13	1	1	0.747
Edema bone<br>+ Matr. Calcifications <2/3	Yes<br>No	4<br>19	0<br>59	27.46 (1.41–533.26)	0.029	0.174	1	1	0.747
Edema bone<br>+ Endosteal scalloping (any)	Yes<br>No	4<br>19	0<br>59	27.46 (1.41–533.26)	0.029	0.174	1	1	0.747
Edema bone<br>+ Endosteal scalloping > 2/3	Yes<br>No	4<br>19	0<br>59	27.46 (1.41–533.26)	0.029	0.174	1	1	0.747
Edema bone<br>+ Penetration	Yes<br>No	2<br>21	0<br>59	13.83 (0.64–299.94)	0.094	0.087	1	1	0.737
Edema soft tissue<br>+ Expansion	Yes<br>No	2<br>21	0<br>59	13.84 (0.64–299.94)	0.094	0.087	1	1	0.737
Edema soft tissue<br>+ Matr. Calcifications <1/3	Yes<br>No	5<br>18	0<br>59	35.37 (1.87–670.38)	0.018	0.217	1	1	0.766/

Edema soft tissue + Matr. Calcifications <2/3	Yes<br>No	6<br>17	1<br>58	17.68 (2.66–480.45)	0.002	0.261	0.983	0.857	0.773
Edema soft tissue + Endosteal scalloping (any)	Yes<br>No	6<br>17	0<br>59	44.2 (2.37–824.01)	0.011	0.261	1	1	0.776
Edema soft tissue + Endosteal scalloping > 2/3	Yes<br>No	5<br>18	0<br>59	35 (1.87–670.38)	0.018	0.217	1	1	0.766
Edema soft tissue + Penetration	Yes<br>No	4<br>19	0<br>59	27.46 (1.41–533.26)	0.029	0.174	1	1	0.747
Edema bone + Edema soft tissue	Yes<br>No	3<br>20	0<br>59	20.32 (1.01–410.31)	0.05	0.13	1	1	0.747
Extraoss. soft tissue comp. + Edema bone	Yes<br>No	1<br>22	0<br>59	7.93 (0.31–202.0)	0.21	0.044	1	1	0.728
Extraoss. soft tissue comp. + Edema soft tissue	Yes<br>No	1<br>22	0<br>59	7.93 (0.31–202.0)	0.21	0.044	1	1	0.728

Odds ratio, Sensitivity, Specificity, PPV and NPV given for the identification of ACT versus enchondroma, respectively. PPV = positive predictive value; NPV = negative predictive value; CI = confidence interval. Values of the more senior reader are given.

Table S5. Analysis of combinations of features with high sensitivity and features with high specificity.

Combined Sens + Spec		ACT	Ench.	Oddsratio (95% CI)	P-value	Sensitivity	Specificity	PPV	NPV
Matr. Calcifications <2/3	Yes	8	2	13.87 (3.01–109.46)	<0.001	0.348	0.966	0.8	0.792
+ Periosteal reaction	No	15	57						
Matr. Calcifications <2/3	Yes	10	9	4.17 (1.39–12.89)	0.01	0.435	0.847	0.526	0.794
+ Expansion	No	13	50						
Matr. Calcifications <2/3	Yes	14	5	15.67 (4.76–60.78)	<0.001	0.609	0.915	0.737	0.857
+ Endosteal scalloping > 2/3	No	9	54						
Cont. growth pattern	Yes	6	1	17.68 (2.66–480.45)	0.002	0.261	0.983	0.857	0.773
+ Endosteal scalloping (any)	No	17	58						
Endosteal scalloping (any)	Yes	8	2	13.87 (3.01–109.46)	<0.001	0.348	0.966	0.8	0.792
+ Periosteal reaction	No	15	57						
Endosteal scalloping (any)	Yes	10	10	3.69 (1.25–11.12)	0.02	0.435	0.831	0.5	0.79
+ Expansion	No	13	49						

Odds ratio, Sensitivity, Specificity, PPV and NPV given for the identification of ACT versus enchondroma, respectively. PPV = positive predictive value; NPV = negative predictive value; CI = confidence interval. Values of the more senior reader are given.
